# Impact of smoking on procedural outcomes and all-cause mortality following acute myocardial infarction: A misleading early-stage pseudoparadox with ultimately reduced survival

**DOI:** 10.1016/j.ijcrp.2024.200336

**Published:** 2024-09-27

**Authors:** Mohammed Abusharekh, Jürgen Kampf, Iryna Dykun, Viktoria Backmann, Rolf Alexander Jánosi, Matthias Totzeck, Tienush Rassaf, Amir Abbas Mahabadi

**Affiliations:** Department of Cardiology and Vascular Medicine, The West German Heart and Vascular Center Essen, University Hospital Essen, 45147, Essen, Germany

**Keywords:** Myocardial infarction, NSTEMI, Smoker's paradox, Smoking, STEMI, TIMI flow

## Abstract

**Background:**

Smoking has conflicting results on outcomes following acute myocardial infarction (AMI). We evaluated the independent influence of smoking status on patient outcomes.

**Methods:**

We included patients with AMI undergoing invasive coronary angiography with available self-reported smoking status. The incidence of death of any cause was evaluated during a median follow-up of 1.14 years (range 0.36–3.40 years). Association between smoking status and long-term mortality was evaluated using multivariable adjusted Cox regression analysis.

**Results:**

From 1612 AMI patients (aged 65.7 ± 13.3 years, 72.1 % male), 378 patients (23.4 %) were current-smokers, 311 (19.3 %) ex-smokers, and 923 (57.3 %) non-smokers. Compared with non-smokers, current-smokers were younger (68.5 ± 13.0 vs. 58.6 ± 12.5, p < 0.0001) and more frequently presented with STEMI (21.6 % vs. 35,4 %, p < 0.0001), while ex-smokers with similar frequency of STEMI-manifestation as non-smokers (22.5 %, p = 0.79) constituted an intermediate-group in terms of age (65.8 ± 11,6 years). Although smoking status was not significantly associated with long-term survival in unadjusted-analysis, active-smokers had 56 % higher long-term mortality than non-smokers when adjusting for age, gender, medications and other traditional risk factors, whereas ex-smokers possessed comparable survival probability (current-smokers: 1.56[1.14–2.14], p = 0.006, ex-smokers 1.16[0.84–1.59], p = 0.37). Current-smokers had unadjusted lower NT-proBNP and modestly higher absolute in-hospital left ventricular global longitudinal strain (LV GLS) values that did not differ among groups after the same adjustments (NT-proBNP: −0.08[-0.31; 0.15], p = 0.5, LV GLS: 0.65[-0.26; 1.55], p = 0.16).

**Conclusion:**

Active smoking is associated with increased adjusted long-term mortality, earlier onset and more frequent manifestation as STEMI, compared to non-smoking. Comparable adjusted results for LV GLS and NT-proBNP between groups support the presence of the pseudoparadox.

## Highlights

• Smoking poses a burden of morbidity and mortality for acute myocardial infarction.
• Conflicting evidence for the existence of a short-term smokers' paradox is debated.
• Smoking reduces survival and leads to younger presentation with myocardial infarction.
• Favorable longitudinal strain and NT-proBNP in smokers are due to a low risk profile.
• Our data suggest a pseudo-paradox, as smokers are younger and have less comorbidities.

## Graphical abstract

**Figure:**
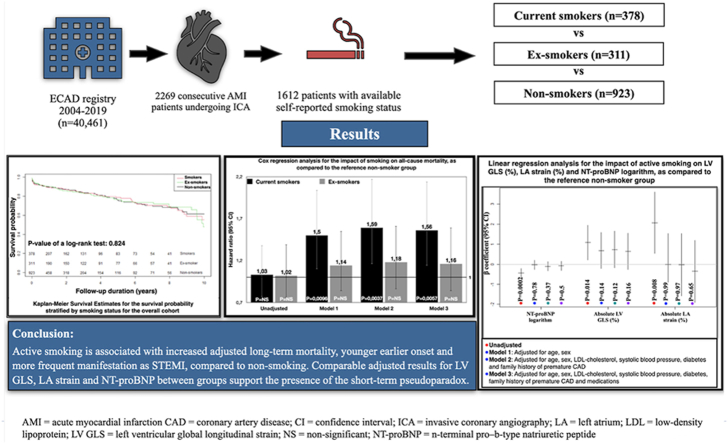

## 1 Introduction

Smoking, which is known to be associated with an increased risk for atherosclerotic cardiovascular disease (ASCVD), including acute myocardial infarction (AMI), is responsible for half of all preventable deaths [1]. Regardless of the age of the first cardiovascular event, there is accumulating evidence that smoking cessation significantly reduces the risk of all-cause death, and is associated with a better prognosis in terms of recurrent major atherosclerotic cardiovascular events (MACE), making smoking cessation an integral part of the guideline-directed prevention therapy [2]. However, the occasionally observed association between smoking and favorable short-term outcomes after many cardiovascular diseases, including AMI, known in the literature as the "smoker's paradox” has been a matter of noteworthy debate for years [3].

This paradox, first documented in previous studies on the prognosis of AMI and later confirmed by studies in the era of thrombolysis, has also been described in more recent data in patients with ST-elevation myocardial infarction (STEMI), non-ST-elevation myocardial infarction (NSTEMI), cardiac arrest and acute ischemic stroke [[4], [5], [6], [7]]. On the other hand, there is conflicting evidence suggesting that this phenomenon is a fallacy, attributing it to the fact that smokers are significantly younger when they experience their first AMI and therefore have a lower burden of cardiovascular risk factors and comorbidities than non-smokers [8,9]. Likewise, any modest improvement in short-term outcomes, albeit controversial, is reversed by a significant increase in long-term morbidity and mortality [10].

Considering that smoking status, which is closely related to overall patient's health as well as risk factor profile, has conflicting effects on short-term outcomes following AMI in the literature, we aimed to investigate how it affects both short-term procedural outcomes and long-term mortality, using appropriate risk factor adjustments. At the same time, to further elucidate the influence of smoking on left ventricular function and heart failure development following acute myocardial infarction, we planned to reveal the potential differences in speckle tracking echocardiography measurements as well as natriuretic peptides in a large cohort of acute coronary syndromes.

## 2 Methods

### 2.1 Study cohort

The present analysis was conducted using the longitudinal ECAD (Essen Coronary Artery Disease) registry cohort of patients who underwent invasive conventional coronary angiography at the West German Heart and Vascular Center Essen in the period from 2004 to 2019. The ECAD registry comprises data from 40,461 coronary artery procedures (as of July 2019). In the current analysis, we enrolled patients whose primary discharge diagnosis corresponded to STEMI or NSTEMI, on the basis of the International Statistical Classification of Diseases (ICD 10). Patients with lack of follow-up information or smoking status documentation or with missing discharge diagnosis were ruled out, yielding a final analysis cohort of 1612 patients with AMI (403 patients as STEMI, 1208 patients as NSTEMI, one patient was classified as neither STEMI nor NSTEMI). Approval for the presented analysis was granted by the local ethics committee (19-8956-BO).

### 2.2 Clinical features, procedural characteristics and covariate evaluation

Data on standard cardiovascular risk factors during the same hospital stay were retrieved from the hospital information system and subsequently integrated into the database. Laboratory parameters were assessed by means of standardized enzymatic methods [low- and high-density lipoprotein (LDL-, HDL-) cholesterol, lipoprotein (LP(a))]. Diabetes mellitus was defined as HbA1c value of ≥6.5 %. Systolic blood pressure measurements, self-proclaimed statements regarding smoking behavior and family anamnesis of premature coronary artery disease (CAD) were recorded. Relevant information on medications were drawn from the discharge prescriptions and were restricted to 1425 cases. Non-statin lipid-lowering therapy was determined as treatment with ezetimibe, fibrate, bile acid sequestrates, niacin and/or PCSK-9 inhibitors. Oral anticoagulants encompassed the direct oral anticoagulants (DOAC), i.e. dabigatran, rivaroxaban, edoxaban and apixaban, as well as vitamin K antagonists (phenprocoumon or warfarin). P2Y12 inhibitors included clopidogrel, prasugrel and ticagrelor. The information on age and gender was also obtained from the hospital information system. Assessment of all invasive coronary angiography (ICA) procedures including the interpretation of thrombolysis in myocardial infarction (TIMI) flow was conducted in offline settings at the central imaging core laboratory of the West German Heart and Vascular Center by a highly dedicated interventional cardiologist who was blinded to the patient's clinical presentation and all other variables recorded in the ECAD registry. A final angiographic TIMI flow grade ≤2 in the infarct-related coronary artery in the absence of evidence for dissection, obstruction, thrombus, or spasm at the time of termination of the percutaneous coronary intervention was defined as no-reflow phenomenon (NRP) [11]. Cardiogenic shock (CS) was defined as fulfilling the criteria established by the Society for Cardiovascular Angiography and Interventions (SCAI) for stage B, C, D or E cardiogenic shock (i.e. persistent systolic blood pressure less than 90 mmHg or mean arterial pressure less than 60 mmHg or a fall of more than 30 mmHg from the baseline value and utilization of vasopressors and/or mechanical circulatory support for maintaining blood pressure higher than these targets) [12]. Acute total occlusion (ATO) was described as the acute presence of a TIMI flow of 0–1 in the artery associated with the infarction or a TIMI flow of 2–3 with concomitant severely elevated peak troponin levels (>100 times the upper reference value), corresponding to the large infarct area compatible with occlusion, as previously defined [13]. Right dominance was determined as the posterolateral branch (PLB) as well as the posterior descending artery (PDA) arising from the right coronary artery (RCA), left dominance when both branches originate from the left circumflex artery (LCx), and codominance when the PDA arises from the RCA and the PLB from the LCx [14].

### 2.3 Echocardiographic assessment of myocardial deformation and systolic functions

Existing transthoracic echocardiographic examinations (TTE) following ICA with satisfactory image resolution (n = 864) were analyzed offline in the imaging core laboratory of the West German Heart and Vascular Center by specially trained study personnel who were blinded to the patients' clinical presentations, covariates and coronary angiography findings. The end-systolic volume (ESV) and end-diastolic volume (EDV) of the left ventricle (LV) were acquired with manual tracing in apical four- and two-chamber views and calculated according to the Simpson method (Biplane). When a biplane evaluation was not feasible, LVESV and LVEDV were assessed in a four-chamber view alone. Likewise, the strain measurement was conducted offline utilizing commercially available software (TOMTEC-Arena 2D Cardiac Performance Analysis) as outlined previously. Briefly, the endocardial boundaries were manually traced in the end-systolic image and automatically tracked by the software over the entire cardiac cycle. Minor adjustments to the end-diastolic frame were applied manually if necessary. LV global longitudinal strain (LV GLS) was subsequently computed by the software utilizing apical two-, three-, and four-chamber views. For the calculation of left atrial (LA) strain, apical two- and four-chamber views were employed, while the apical four-chamber view was used to determine right ventricular (RV) strain.

### 2.4 Endpoint definition

Mortality from all causes was defined as the primary endpoint variable. Survival information was obtained from all available hospital records (including partner healthcare facilities) and insurance records. Any outpatient or inpatient admission to the West German Heart and Vascular Center, Essen University Hospital or any partner facility following a coronary investigation was used to verify survival status. Patients without confirmed mortality but no subsequent referrals to the healthcare provider were regarded as missing at follow-up and were excluded from the present analysis.

### 2.5 Statistical analysis

The baseline characteristics are presented as the mean ± standard deviation for normally distributed continuous variables, as the median and interquartile range for ordinal or skewed continuous variables, and as frequency and percentages for categorical variables. Two-sided t-tests were used for normally distributed continuous variables, Wilcoxon tests were applied to ordinal or skewed continuous variables and chi-square tests were used for categorical variables for pairwise comparison of the 3 subgroups of the study (current smokers, ex-smokers and non-smokers). The incidence of death from any cause during follow-up was recorded. Kaplan-Meier analysis was used to depict the survival probability in study groups, stratified by smoking status. Cox regression analysis was used to determine the association of smoking with incident mortality. Linear-regression analyses were performed to evaluate the relationship between smoking status and the following variables: NTproBNP, LV GLS and LA strain. Adjustment sets were defined as follows (1): unadjusted (2); age and sex-adjusted (3); ancillary adjustment for low density lipoprotein (LDL) cholesterol, systolic blood pressure, diabetes and family history of premature CAD and current smoking status (model 2); and (4) model 2 + medication use (statin, aspirin, P2Y12-inhibitors, antihypertensive medication). In order to further confirm the concept of pseuoparadox, age- and sex-adjusted Kaplan-Meier curves were generated and a short-term survival analysis was conducted, truncating all events at 90 days and using unadjusted and adjusted Cox regression analyses. All analyses were performed using R Statistical Software (version 4.2.0, R Foundation for Statistical Computing, Vienna, Austria). A p-value <0.05 indicated statistical significance.

## 3 Results

Overall, 1612 patients (mean age 65.7 ± 13.3 years, 72.1 % male) with acute myocardial infarction were included in our analysis. Of these, 378 patients (23.4 %) were current smokers, 311 patients (19.3 %) were ex-smokers, and the remaining 923 patients (57.3 %) were non-smokers. Compared with non-smokers, current smokers were younger, more frequently male, had lower baseline blood pressure and higher frequency of premature coronary artery disease in their family history. Moreover, they had lower HDL-cholesterol, NT-proBNP and CRP values, higher hemoglobin levels, and both higher admission and peak CK values. Compared with active smokers, ex-smokers were older (but slightly younger than non-smokers), had lower LDL-cholesterol (but higher lipoprotein (a)), and lower peak CK values. No difference was observed between the three groups in terms of diabetes frequency, presence of obesity and the course of troponin values. The baseline characteristics for the overall cohort and stratified by the groups are shown in detail in Table 1.

**Table 1:** Baseline characteristics.

	Overall (n = 1612)	Current smokers (n = 378)	Ex-smokers (n = 311)	Non-smokers (n = 923)	p-value (Current smokers vs. Ex-smokers)	p-value (Current smokers vs. Non-smokers)	p-value (Ex-smokers vs. Non-smokers)
**Demographics**							
Age, years	65.7 ± 13.3	58.6 ± 12.5	65.8 ± 11.6	68.5 ± 13	**< 0.0001**	**< 0.0001**	**0.0006**
Sex, male, n (%)	1163 (72.1)	295 (78)	259 (83.3)	609 (66)	0.1	**< 0.0001**	**< 0.0001**
BMI (kg/m2)	29 ± 10.1	32.8 ± 20.2	29.5 ± 6.02	27.6 ± 5.2	0.4	0.17	0.13
**Cardiovascular risk factors**							
Diabetes mellitus, n (%)	213 (13.2)	42 (11.1)	45 (14.5)	126 (13.7)	0.23	0.25	0.79
Premature family CAD history, n (%)	366 (22.7)	128 (33.9)	104 (33.4)	134 (14.5)	0.97	**< 0.0001**	**< 0.0001**
Systolic blood pressure, mmHg	136 ± 23.8	134 ± 23.3	135 ± 26.3	138 ± 23	0.4	**0.0046**	0.15
**Laboratory measurements**							
LDL, mg/dl	111 ± 41.4	114 ± 41.4	108 ± 39.2	112 ± 42.1	**0.046**	0.49	0.094
HDL, mg/dl	44.5 ± 15.4	42.9 ± 15.4	44.3 ± 15.3	45.3 ± 15.5	0.34	**0.039**	0.39
LP(a), mg/dl	17 (7; 47)	13.5 (7; 43)	21 (9; 58)	17 (7.5; 46)	**0.031**	0.32	0.11
BNP, pg/ml^a^	171 (76.7; 483)	189 (59.7; 663)	118 (70.2; 274)	182 (95.8; 482)	0.17	0.97	**0.042**
NTproBNP, pg/ml^a^	1012(22 0; 4261)	661 (189; 2544)	921 (230; 3846)	1162 (230; 4863)	0.18	**0.025**	0.54
CK (peak), IU/l	239 (116; 780)	302 (128; 1007)	233 (117; 723)	221 (112; 693)	**0.037**	**0.00069**	0.48
CRP, mg/dl	1.3 (0.7; 2.8)	1.2 (0.6; 2.5)	1.1 (0.6; 2.2)	1.45 (0.7; 3.27)	0.52	**0.019**	**0.0035**
Ferritin, ng/ml	177 (83; 318)	194 (107; 357)	184 (74.5; 273)	153 (84; 327)	0.55	0.7	0.72
Hemoglobin, g/dl	13.5 ± 1.93	14.1 ± 1.94	13.5 ± 1.81	13.2 ± 1.92	**< 0.0001**	**< 0.0001**	**0.028**
Percentiles of Troponin (peak), median (Q1; Q3)	0.50 (0.25; 0.75)	0.55 (0.24; 0.78)	0.51 (0.25; 0.75)	0.48 (0.26; 0.74)	0.45	0.21	0.7
Percentiles of Troponin (admission), median (Q1; Q3)	0.50 (0.26; 0.75)	0.49 (0.26; 0.77)	0.51 (0.26; 0.73)	0.50 (0.24; 0.76)	0.56	0.66	0.69
**Medication**							
Statin (n = 1173), n (%)	956 (81.5)	232 (86.6)	195 (89.4)	529 (77)	0.41	**0.0013**	**< 0.0001**
Non-statin cholesterol-lowering therapy (n = 1173), n (%)	39 (3.3)	5 (1.9)	9 (4.1)	25 (3.6)	0.23	0.23	0.9
Beta-blocker (n = 1425), n (%)	1295 (90.9)	315 (93.5)	255 (91.7)	725 (89.5)	0.5	**0.046**	0.34
Diuretic (n = 1425), n (%)	814 (57.1)	166 (49.1)	171 (61.5)	477 (59)	**0.0027**	**0.0027**	0.5
ACEI or ARB (n = 1423), n (%)	1054 (74.1)	260 (77.2)	201 (72.6)	593 (73.3)	0.22	0.2	0.87
Calcium antagonist (n = 1423), n (%)	383 (26.9)	60 (17.8)	78 (28.1)	245 (30.3)	**0.0033**	**< 0.0001**	0.52
Aspirin (n = 1366), n (%)	1223 (89.5)	309 (95.7)	250 (94)	664 (85.5)	0.46	**< 0.0001**	**0.0004**
P2Y12 inhibitors (1359), n (%)	1040 (76.5)	264 (82)	227 (85.3)	549 (71.2)	0.33	**0.0003**	**< 0.0001**
Oral anticoagulation (n = 1354), n (%)	261 (19.3)	34 (10.6)	58 (21.8)	169 (22.1)	**0.0003**	**< 0.0001**	1

Values of continuous variables are reported as mean ± SD if approximately normally distributed, and median (interquartile range) if not approximately normally distributed. Categorical variables are reported as n (%).

ACEI = angiotensin-converting enzyme inhibitor; ARB = angiotensin II receptor blocker; BNP = brain natriuretic peptide; CAD = coronary artery disease; CK = creatine kinase; CRP = c-reactive protein; HDL-C = high-density lipoprotein cholesterol; LDL-C = low-density lipoprotein cholesterol; LP (a) = Lipoprotein (a); NT-proBNP = n-terminal pro–b-type natriuretic peptide.

a BNP values available in 156 patients, NT-proBNP values available in 675 patients.

### 3.1 Smoking status and pharmacotherapy management

Active smokers received more frequently statins, aspirin, P2Y12 inhibitors, and beta-blockers compared to non-smokers. However, smokers were less likely to be treated with calcium channel antagonists, diuretics, and oral anticoagulation treatments compared to the other two groups in which no difference was observed (Table 1).

### 3.2 Smoking status and periprocedural characteristics

Presence of ST elevation on standard ECG and angiographically detection of acute total occlusion were observed more frequently in active smokers than in non-smokers. Similarly to active smokers, ex-smokers were associated with more acute occlusion but less presentation with STEMI, comparable to non-smokers. Likewise, active smoking was observed to be associated with a less favorable preprocedural TIMI flow than never smoking. While no difference was observed between the groups in terms of TIMI flows following the intervention. There was no difference between the groups in terms of infarct-related artery location, and the incidence of cardiogenic shock and no reflow, as well as the number of stents implanted during the procedure were comparable between groups (Table 2).

**Table 2:** Procedural characteristics.

Angiographic characteristics	Overall (n = 1612)	Current smokers (n = 378)	Ex-smokers (n = 311)	Non-smokers (n = 923)	p-value (Current smokers vs. Ex-smokers)	p-value (Current smokers vs. Non-smokers)	p-value (Ex-smokers vs. Non-smokers)
Clinical presentation, STEMI, n (%)	403 (25)	134 (35.4)	70 (22.5)	199 (21.6)	**0.0003**	**< 0.0001**	0.79
Acute total occlusion, n (%)	575 (35.7)	161 (42.6)	123 (39.5)	291 (31.5)	0.47	**0.0002**	**0.012**
No-reflow Phenomenon, n (%)	26 (1.62)	6 (1.59)	4 (1.29)	16 (1.74)	0.99	1	0.78
Cardiogenic shock, n (%)	24 (1.49)	6 (1.59)	4 (1.29)	14 (1.52)	0.99	1	0.98
TIMI before intervention, n (%)					0.097	**0.0083**	0.61
0–2	375 (23.3)	104 (27.5)	73 (23.5)	198 (21.6)			
3	1231 (76.7)	274 (72.5)	238 (76.5)	719 (78.4)			
TIMI after intervention, n (%)					0.6	0.2	0.57
0–2	132 (8.2)	35 (9.3)	29 (9.3)	68 (7.4)			
3	1476 (91.8)	343 (90.7)	282 (90.7)	851 (92.6)			
Number of implanted stents, n (%)					0.9	0.093	0.09
<2	1275 (79.2)	295 (78)	247 (79.4)	733 (79.6)			
≥2	335 (20.8)	83 (22)	64 (20.6)	188 (20.4)			
Infarct related artery, n (%)					0.57	0.22	0.62
Left main coronary artery	76 (4.71)	12 (3.17)	17 (5.47)	47 (5.09)			
Left anterior descending artery	441 (27.4)	112 (29.6)	83 (26.7)	246 (26.7)			
Circumflex artery	219 (13.6)	61 (16.1)	47 (15.1)	111 (12)			
Right coronary artery	312 (19.4)	73 (19.3)	57 (18.3)	182 (19.7)			
Not determined	564 (35)	120 (31.7)	107 (34.4)	337 (36.5)			
Coronary artery dominance					**0.017**	**0.0036**	0.94
Right	699 (43.4)	145 (38.5)	138 (44.4)	416 (45.2)			
Left	638 (39.7)	149 (39.5)	128 (41.2)	361 (39.2)			
Co-dominance	272 (16.9)	83 (22)	45 (14.5)	144 (15.6)			

Values of continuous variables are reported as mean ± SD if approximately normally distributed, and median (interquartile range) if not approximately normally distributed. Categorical variables are reported as n (%).

STEMI = ST-Elevation Myocardial Infarction; TIMI = thrombolysis in myocardial infarction.

### 3.3 Active smoking and long-term survival

In unadjusted Cox regression analysis as well as in Kaplan-Meier analysis, no difference was observed between the groups in terms of long-term survival during a median follow-up of 1.14 years (range 0.36–3.40 years, up to 14.60 years) (Fig. S1). However, when adjusting for age and sex, active smoking was linked with a 50 % increased hazard ratio of mortality. After adjusting for age, sex, other conventional cardiovascular risk factors, and medications, effect sizes persisted with active smoking being associated with a 56 % increase in mortality compared to the reference non-smoker group, while no difference persisted in the ex-smoker group (Table 3, Fig. S2).

**Table 3:** Cox regression analysis for the impact of smoking on all-cause mortality.

	Non-smokers (reference)		Current smokers		Ex-smokers	
	Hazard ratio (95 % CI)	p-value	Hazard ratio (95 % CI)	p-value	Hazard ratio (95 % CI)	p-value
Unadjusted	1.0	–	1.03 (0.77–1.38)	0.84	1.02 (0.76–1.39)	0.88
Model 1	1.0	–	1.50 (1.10–2.04)	**0.0096**	1.14 (0.84–1.55)	0.406
Model 2	1.0	–	1.59 (1.16–2.17)	**0.0037**	1.18 (0.86–1.61)	0.299
Model 3	1.0	–	1.56 (1.14–2.14)	**0.0057**	1.16 (0.84–1.59)	0.365

Model 1: Adjusted for age, sex.

Model 2: Adjusted for age, sex, LDL-cholesterol, systolic blood pressure, diabetes and family history of premature CAD.

Model 3: Adjusted for age, sex, LDL-cholesterol, systolic blood pressure, diabetes, family history of premature CAD and medications.

CAD = coronary artery disease; CI = confidence interval; LDL = low-density lipoprotein.

### 3.4 Active smoking and short-term survival

Unadjusted Cox regression analysis and Kaplan-Meier curve showed no difference between groups with respect to short-term survival at 90 days (Fig. S3). However, when adjusting for age and sex, active smoking was associated with a 75 % increased hazard ratio of mortality. After adjusting for age, sex, and other cardiovascular risk factors, effect sizes maintained with active smoking being associated with an 81 % increase in mortality compared to the reference non-smoker group, while no difference remained in the ex-smoker group (Table S1, Fig. S4).

### 3.5 Active smoking and systolic left ventricular function after myocardial infarction

**Fig. 1:**
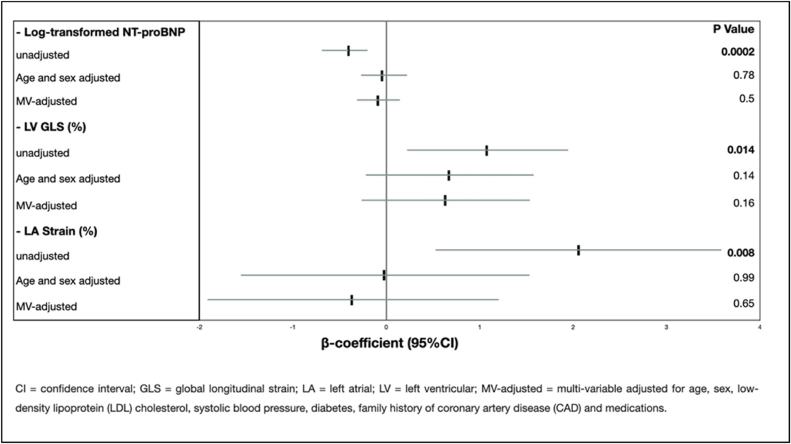
Linear regression analysis for the association of active smoking on LV GLS (%), LA strain (%) and log-transformed NT-proBNP as continuous variables, using non-smokers as reference.

Aiming to provide more comprehensive insights into the effects of active smoking, we compared LV GLS, RV GLS, and LA strain in addition to LV EF measurements between all three groups. While no difference was observed concerning LVEF and RV GLS, slightly more favorable LV GLS and LA strain measurements were found in active smokers (Table S2). However, in a linear regression analysis after adjusting for age, sex, other traditional cardiovascular risk factors, and medications, no difference was observed. Likewise, the observed difference in NT-proBNP in active smokers disappeared after the same adjustments (Fig. 1).

### 3.6 Association between smoking and periprocedural TIMI flow in STEMI vs. NSTEMI subgroups

In the subgroup analysis, no correlation was observed between smoking and periprocodural TIMI flow in patients presenting with STEMI, whereas active smokers in the NSTEMI subgroup were associated with less farvorable TIMI flow both before and after the intervention as compared to non-smokers (Table S3).

## 4 Discussion

In the present large longitudinal observational registry of consecutive acute myocardial infarction patients undergoing coronary angiography, we demonstrate that (I) active smoking is associated with both a significantly younger age of presentation and a more frequent manifestation as ST-elevation myocardial infarction as well as angiographic detection of acute total occlusion as compared to non-smoking; (II) although smoking status does not appear to have an effect on unadjusted long-term survival, active smokers have higher long-term mortality than both ex-smokers and non-smokers when adjusting for age, gender, and other potential confounding factors (III); unadjusted NT-proBNP, LV GLS and LA strain values, which seem to be protective in favor of current smokers, did not differ among groups after the same adjustments, supporting the concept of pseudoparadox in the short term. Our results therefore not only clarify that active smoking is not associated with favorable short-term outcomes, but also leads to worse preprocedural TIMI flow, earlier age at presentation of AMI, and reduced long-term survival.

Smoking is a well-known risk factor for acute myocardial infarction (AMI), which constitutes a significant burden of mortality worldwide and is one of the leading risk factors for disability-adjusted life years [1,15]. However, a more favorable prognosis in active smokers has been postulated by some post-AMI short-term studies and has been reported in the literature as the smoker's paradox [[3], [4], [5]]. Various mechanisms have been proposed to elucidate this paradox; some of these are lower platelet reactivity index independent of antithrombotic agent, reducing ischemia-reperfusion injury and infarct size by enhancing hypoxia-related preconditioning pathways and likewise the chronic inflammatory preconditioning [[16], [17], [18]]. On the contrary, and in accordance with the evidence refuting this paradox, our results emphasize the fact that smokers present with AMI at a younger age (approximately 10 years) and therefore with less comorbidities [8,9]. Many observational studies that support the smoker's paradox lack the adjustment for confounding factors, particularly age. Furthermore, many of these observations are based on post hoc analyzes of randomized controlled trials of the thrombolytic era reflect a highly selected patient group that not fully represent the current practice [19,20]. The present analysis is consistent with studies that consider the appropriate adjustment of risk factors and ultimately identify cigarette-related deleterious rather than protective effects. Compared to most other previous studies, our study has a long follow-up of up to 15 years and covers the entire AMI spectrum, revealing insights to perceive this discrepancy from an inclusive perspective [9,21].

One of the well-documented early complications of AMI is the phenomenon of no reflow, characterized by myocardial tissue hypoperfusion despite a patent epicardial coronary artery following intervention and has been shown to be closely associated with reduced in-hospital, short- and long-term survival [22,23]. Some reports have suggested that in a significant proportion of smokers the histological composition of the culprit lesion is thrombogenic rather than atherogenic, affecting the natural course of STEMI, tending to be more susceptible to antithrombotic therapy, mechanical or fibrinolytic reperfusion, and/or spontaneous lysis, and resulted in better final TIMI flow [[24], [25], [26]]. However, in agreement with the contrary studies in the literature, we observed no difference in final TIMI flow in our STEMI patient subgroup, possibly due to the greater thrombus burden and distal embolization risk in smokers [27,28]. Moreover, our finding of the more commonly low baseline TIMI flow in the NSTEMI subgroup is aligned with the ACUITY (Acute Catheterization and Urgent Intervention Triage Strategy) trial's large-scale analysis in this regard. However, unlike this analysis, we observed even slightly lower postprocedural TIMI flow for active smokers in NSTEMI, eliminating any beneficial effect of smoking on coronary flow dynamics [29].

Infarct size is an important independent predictor of long-term survival and the development of heart failure after AMI [30]. It has been claimed that active smoking may limit infarct size by reducing ischemia-reperfusion injury through preconditioning of the myocardium [21,31]. However, in our study, we found that the enzymatic infarct size (indicated by creatine kinase) enlarged rather than decreased in active smokers, as compared to both ex- and non-smokers. Likewise, recent cardiac magnetic resonance studies have also disproved this hypothesis. They revealed a higher incidence of myocardial hemorrhage reflecting irreversible microvascular bed damage at the infarct site in smokers, providing insights for the association of smoking with adverse left ventricular remodelling and worse long-term prognosis [26,31].

Limited evidence regarding the impact of coronary artery dominance on prognosis after acute coronary syndromes revealed that left and codominance are associated with modestly greater in-hospital mortality [14]. This finding was predominantly attributed to the greater proportion of myocardium at risk in the event of obstruction in this less well-balanced non-right dominant coronary anatomy. In addition, left coronary dominance in STEMI has also been reported to adversely affect long-term survival, even after accounting for the more common observed left ventricular dysfunction [32]. The greater non-right dominance we observed in current smokers compared to the other two groups may have contributed to the worse long-term prognosis. However, whether a more intensive treatment strategies should be pursued in smokers without right coronary dominance remains to be addressed prospectively.

### 4.1 Clinical implications

The results of our study have direct implications for clinical practice and public health. Herein, we provide evidence that most procedural outcomes are comparable in non-smokers and ex-smokers and that they also have similar long-term survival following AMI. Furthermore, with our findings demonstrating increased long-term mortality associated with active smoking and disproving the ‘smoker's paradox’ in AMI, we underscore the critical importance of clinicians recommending smoking cessation initiatives to their patients. In the same context, contemporary evidence in the literature as well as the present results indicate that AMI-free life expectancy can be extended by up to 10 years with smoking cessation [10]. Our results also provide insights in terms of reflection on speckle tracking echocardiography, which is becoming increasingly important in clinical practice, and are consistent with the literature highlighting the importance of imaging in acute coronary syndrome [[33], [34], [35], [36]].

### 4.2 Study limitations

Our study findings need to be interpreted taking into account several limitations. Firstly, the ECAD registry is a longitudinal data collection of consecutive patients undergoing invasive coronary angiography. Considering the unbounded inclusion of patients and the observational study design, there are inherent differences in baseline characteristics and treatment profiles among the 3 groups. Secondly, as per study design, missing data and confounding factors that were not accounted for in the regression analysis adjustment may have influenced our results. However, the observed effect sizes were very stable and changed only marginally with additional adjustment for the effect of different medication patterns alongside established cardiovascular risk factors. Thirdly, we recognize that this is a single-center study in which the Caucasian male population constituted the majority. Although, in many points of view, it is in accordance with the previous evidence in the literature and the database includes coronary angiography procedures performed by 74 different interventional cardiologists over a 15-year period, our findings need to be verified both in other centers and healthcare systems and in various ethnic groups. Fourthly, a limitation of the study was that the ECAD database did not include information on duration of smoking, cessation time, and number of cigarettes consumed, therefore, we could not disclose how the potential dose-response relationship would affect the results. However, it has been shown that even smoking one cigarette a day continues to have harmful effects and has about half the risk of coronary artery disease associated with smoking 20 cigarettes a day [37]. Moreover, the results of our study may not be interpreted as directly comparable with all other studies due to the different definitions, since according to the WHO definition, it requires 12 months of quitting time to qualify a smoker as former smoker and our data was derived from discharge letters and accordingly does not correspond to this definition [38]. Finally, it must be acknowledged that the exclusion of patients with missing follow-up information or smoking status and those with missing discharge diagnoses may represent a potential source of selection bias and should be regarded as a limitation of the study.

## 5 Conclusion

In a cohort covering the entire spectrum of acute myocardial infarction, active smoking is associated with decreased adjusted survival, significantly younger presentation (∼1 decade), more frequent manifestation as ST-elevation myocardial infarction in addition to leading to worse preprocedural TIMI flow, compared to non-smoking. Comparable adjusted results for LV GLS, LA strain, and NT-proBNP among groups along with lower adjusted short-term survival in active smokers confirm the short-term pseudo-rationale, indicating that its favorable appearance is predominantly attributable to younger age and lower risk profile.

## Data availability

The data underlying this article will be shared on reasonable request to the corresponding author subject to approval of institutional review boards.

## CRediT authorship contribution statement

**Mohammed Abusharekh:** Writing – review & editing, Writing – original draft, Methodology, Investigation, Formal analysis. **Jürgen Kampf:** Validation, Formal analysis, Data curation. **Iryna Dykun:** Visualization, Conceptualization. **Viktoria Backmann:** Validation, Methodology. **Rolf Alexander Jánosi:** Methodology, Investigation. **Matthias Totzeck:** Data curation, Conceptualization. **Tienush Rassaf:** Supervision. **Amir Abbas Mahabadi:** Writing – review & editing, Validation, Supervision, Formal analysis, Conceptualization.

## Declaration of competing interest

None.
