# Supplementary material for: Impact of smoking on procedural outcomes and all-cause mortality following acute myocardial infarction: A misleading early-stage pseudoparadox with ultimately reduced survival
Source: Int J Cardiol Cardiovasc Risk Prev. 2024 Sep 27;23:200336. doi: 10.1016/j.ijcrp.2024.200336 (PMC11471478; doi:10.1016/j.ijcrp.2024.200336)
Supplement: Multimedia component 1 [file mmc1.docx]

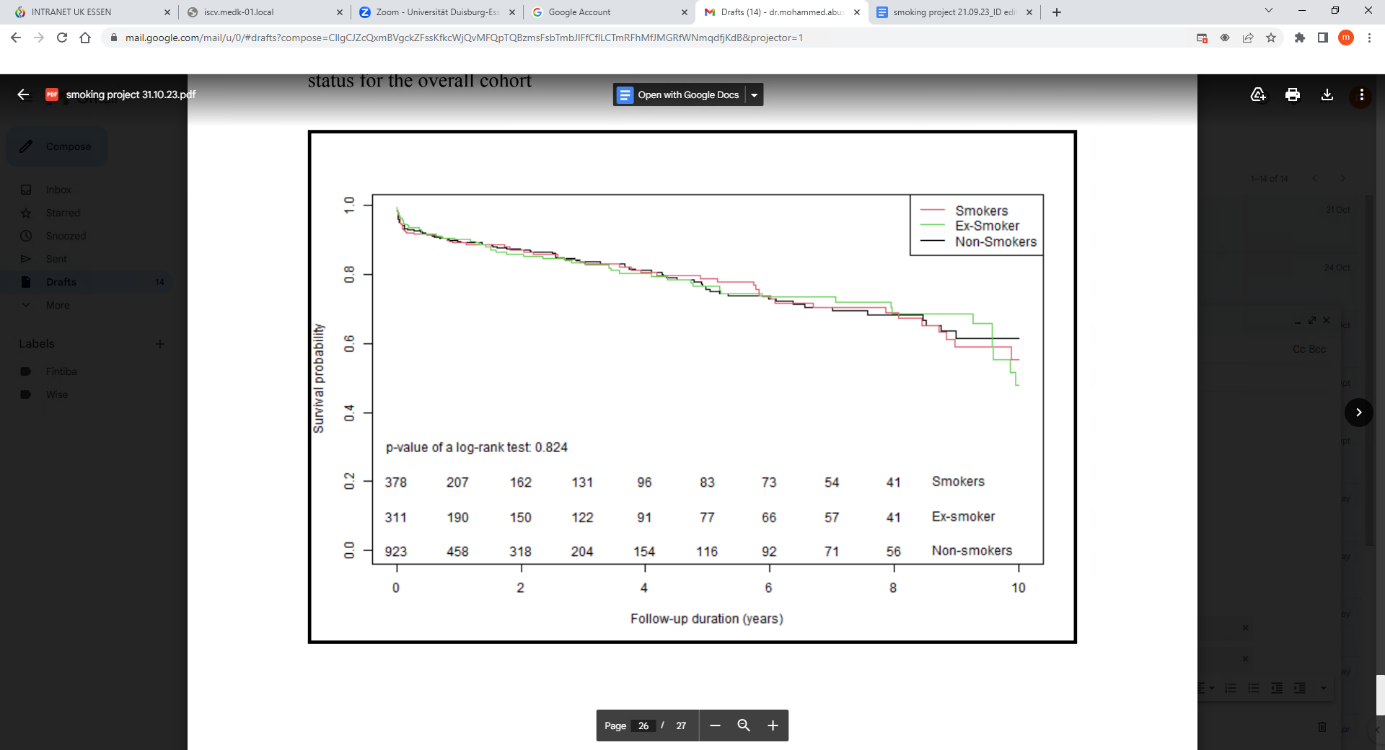
**Figure** **S1**: Kaplan-Meier Survival Estimates for the survival probability stratified by smoking status for the overall cohort

**Figure S2**: Age- and sex-adjusted Kaplan-Meier Survival Estimates for the survival probability stratified by smoking status for the overall cohort


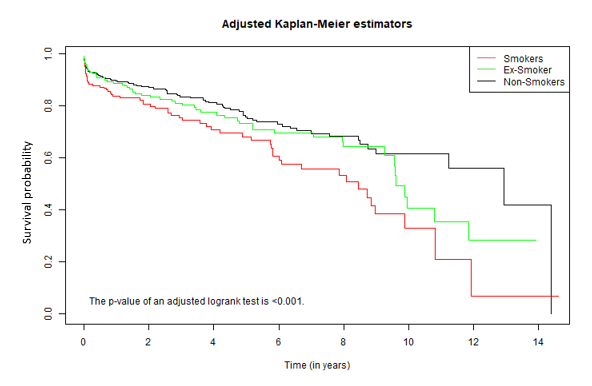


**Figure S3**: Unadjusted Kaplan-Meier Survival Estimates for the short-term survival probability (at 90 days) stratified by smoking status for the overall cohort


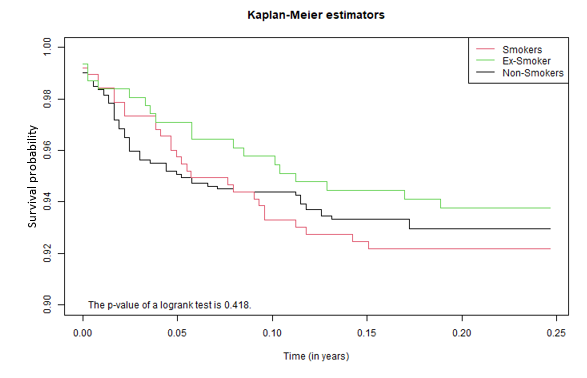


**Figure S4**: Age- and sex-adjusted Kaplan-Meier Survival Estimates for the short-term survival probability (at 90 days) stratified by smoking status for the overall cohort


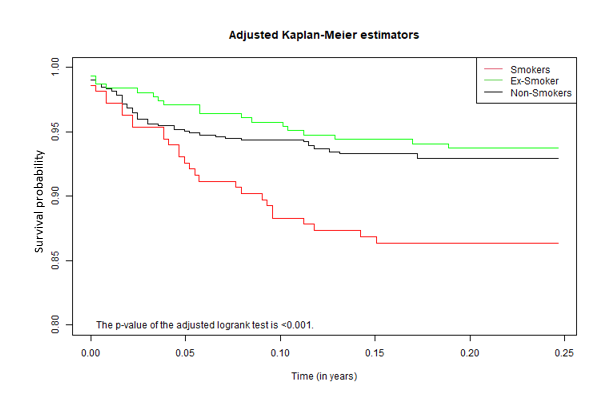


**Table S1:** Cox regression analysis for the impact of smoking on short-term all-cause mortality (at 90 days)

|  | Non-smokers (reference) | | Current smokers | | Ex-smokers | |
| --- | --- | --- | --- | --- | --- | --- |
|  | Hazard ratio (95% CI) | p-value | Hazard ratio (95% CI) | p-value | Hazard ratio (95% CI) | p-value |
| Unadjusted | 1.0 | --- | 1.10 (0.71 - 1.71) | 0.664 | 0.87 (0.52 - 1.45) | 0.589 |
| Model 1 | 1.0 | --- | 1.75 (1.09 - 2.79) | **0.0199** | 1.01 (0.60 - 1.70) | 0.986 |
| Model 2 | 1.0 | --- | 1.81 (1.13 - 2.90) | **0.0133** | 1.06 (0.63 - 1.80) | 0.827 |
| Model 1: Adjusted for age, sex  Model 2: Adjusted for age, sex, LDL-cholesterol, systolic blood pressure, diabetes and family history of premature CAD  CAD = coronary artery disease; CI = confidence interval; LDL = low-density lipoprotein. | | | | | | |

**Table S2**: Echocardiographic parameters by smoking status

| **Echocardiographic parameter** | Overall (n=864) | Current smokers | Ex-smokers | Non-smokers | p-value (Current smokers vs. Ex-smokers) | p-value (Current smokers vs. Non-smokers) | p-value (Ex-smokers vs. Non-smokers) |
| --- | --- | --- | --- | --- | --- | --- | --- |
| LV EF (%) | 49.4 ± 12.2 | 49.7 ± 12.1 | 49.6 ± 12.5 | 49.2 ± 12.1 | 0.92 | 0.58 | 0.68 |
| LV GLS (%) | -15.8 ± 5.27 | -16.6 ± 5.36 | -15.8 ± 4.79 | -15.4 ± 5.37 | 0.21 | **0.025** | 0.42 |
| LA strain (%) | -20.7 ± 9.2 | -22 ± 8.19 | -21.1 ± 8.99 | -19.9 ± 9.62 | 0.33 | **0.0047** | 0.14 |
| RV GLS (%) | -18.4 ± 5.38 | -19.1 ± 5.02 | -18.1 ± 5.29 | -18.3 ± 5.56 | 0.08 | 0.085 | 0.7 |
| EF = ejection fraction; GLS = global longitudinal strain; LA = left atrial; LV = left ventricular; RV = right ventricular | | | | | | | |

**Table** **S3**: Smoking and periprocedural TIMI flow in STEMI vs. NSTEMI subgroups

| **STEMI** | Overall (n=403) | Current smokers (n=134) | Ex-smokers (n=70) | Non-smokers (n=199) | p-value (Current smokers vs. Ex-smokers) | p-value (Current smokers vs. Non-smokers) | p-value (Ex-smokers vs. Non-smokers) |
| --- | --- | --- | --- | --- | --- | --- | --- |
| **TIMI flow before intervention, n (%)** |  |  |  |  | 0.22 | 0.73 | 0.12 |
| 0-2 | 145 (36) | 49 (36.6) | 20 (28.6) | 76 (38.2) |  |  |  |
| 3 | 258 (64) | 85 (63.4) | 50 (71.4) | 123 (61.8) |  |  |  |
| **TIMI flow after intervention, n (%)** |  |  |  |  | 0.092 | 0.23 | 0.47 |
| 0-2 | 35 (8.7) | 9 (6.7) | 8 (11.4) | 18 (9) |  |  |  |
| 3 | 368 (91.3) | 125 (93.3) | 62 (88.6) | 181 (91) |  |  |  |
| **NSTEMI** | Overall (n=1208) | Current smokers (n=244) | Ex-smokers (n=240) | Non-smokers (n=724) | p-value (Current smokers vs. Ex-smokers) | p-value (Current smokers vs. Non-smokers) | p-value (Ex-smokers vs. Non-smokers) |
| **TIMI flow before intervention, n (%)** |  |  |  |  | 0.67 | **0.022** | 0.077 |
| 0-2 | 230 (19.1) | 55 (22.5) | 53 (22.1) | 122 (17.0) |  |  |  |
| 3 | 972 (80.9) | 189 (77.5) | 187 (77.9) | 596 (83.0) |  |  |  |
| **TIMI flow after intervention, n (%)** |  |  |  |  | 0.11 | **0.023** | 0.83 |
| 0-2 | 97 (8.1) | 26 (10.7) | 21 (8.8) | 50 (6.9) |  |  |  |
| 3 | 1107 (91.9) | 218 (89.3) | 219 (91.2) | 670 (93.1) |  |  |  |
| NSTEMI = Non-ST-elevation myocardial infarction; STEMI = ST-elevation myocardial infarction; TIMI = thrombolysis in myocardial infarction. | | | | | | | |
